# Supplementary material for: Patterns of Hearing Loss in Irradiated Survivors of Head and Neck Rhabdomyosarcoma
Source: Cancers (Basel). 2022 Nov 23;14(23):5749. doi: 10.3390/cancers14235749 (PMC9736087; doi:10.3390/cancers14235749)
Supplement: Supplementary file 1 [file cancers-14-05749-s001.zip › cancers-2000410-supplementary.pdf]

Table S1. Ototoxicity classification systems

**Grade**	**SIOP**	**Muenster**	**CTCAEv4.03**
0	≤20 dB HL at all frequencies	≤10 dB HL at all frequencies	<20 dB HL at all frequencies
1	> 20 dB HL SNHL at > 4 kHz	>10 dB HL ≤20 dB HL at any frequency	> 20 dB HL at 8 kHz
2 (a)	> 20 dB HL SNHL at 4 kHz and above	>20 dB HL ≤ 40 dB HL at ≥4 kHz	> 20 dB HL at 4 kHz and above
2 b		>40 dB HL ≤ 60 dB HL at ≥4 kHz	
2 c		>60 dB HL at ≥4 kHz	
3 (a)	> 20 dB HL SNHL at 2 or 3 kHz and above	>20 dB HL ≤ 40 dB HL at <4 kHz	> 20 dB HL at 3 kHz and above
3 b		>40 dB HL ≤ 60 dB HL at <4 kHz	
3 c		>60 dB HL< 80 dB HL at <4 kHz	
4	> 40 dB HL SNHL at 2 kHz and above	≥80 dB at <4 khz	Audiological indication for cochlear implant

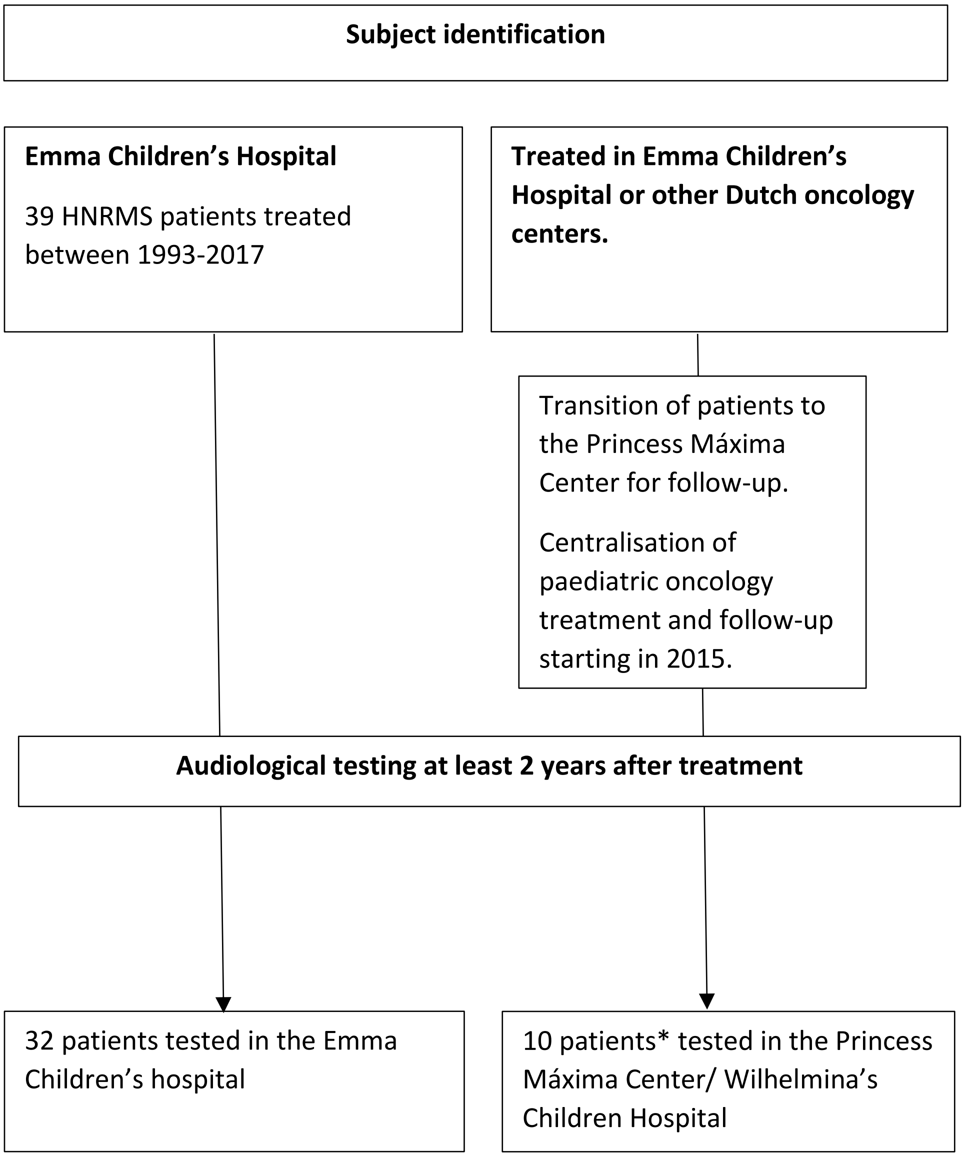

Figure S1. Subject identification flow diagram.
*6 patients were treated in the Emma Children's hospital, 3 at the Sophia's Children hospital and 1 at the University Medical Center Groningen.
